# A Modified Lamb Wave Time-Reversal Method for Health Monitoring of Composite Structures

**DOI:** 10.3390/s17050955

**Published:** 2017-04-26

**Authors:** Liang Zeng, Jing Lin, Liping Huang

**Affiliations:** 1 School of Mechanical Engineering, Xi’an Jiaotong University, Xi’an 710049, China; liangzeng@mail.xjtu.edu.cn (L.Z.); huangliping@stu.xjtu.edu.cn (L.H.); 2 State Key Laboratory for Manufacturing Systems Engineering, Xi’an Jiaotong University, Xi’an 710049, China

**Keywords:** Lamb wave, time reversal, structural health monitoring, composite structures

## Abstract

Because the time reversal operator of Lamb waves varies with frequency in composite structures, the reconstructed signal deviates from the input signal even in undamaged cases. The damage index captures the discrepancy between the two signals without differentiating the effects of time reversal operator from those of damage. This results in the risk of false alarm. To solve this issue, a modified time reversal method (MTRM) is proposed. In this method, the frequency dependence of the time reversal operator is compensated by two steps. First, an amplitude modulation is placed on the input signal, which is related to the excitability, detectability, and attenuation of the Lamb wave mode. Second, the damage index is redefined to measure the deviation between the reconstructed signal and the modulated input signal. This could indicate the presence of damage with better performance. An experimental investigation is then conducted on a carbon fiber-reinforced polymer (CFRP) laminate to illustrate the effectiveness of the MTRM for identifying damage. The results show that the MTRM may provide a promising tool for health monitoring of composite structures.

## 1. Introduction

Composite materials have received extensive attention and have been widely used in high-performance structural applications in a wide range of industries (e.g., aerospace, naval, automotive) because of their high strength, high stiffness-to-weight ratio, and good resistance against aggressive environments. Nevertheless, due to their low transverse strength, composite structures are very sensitive to low-velocity impacts, which can induce a variety of in-service damages, e.g., matrix cracking, delamination, and fiber failure [1,2,3]. These damages can severely degrade mechanical properties and compromise structural integrity [4]. Hence, there is an urgent need for structural health monitoring (SHM) systems to ensure the integrity of composite structures.

With the advantages of fast scanning capabilities, low cost, long-range inspection, and testing inaccessible or complex components, active SHM systems using interrogative Lamb waves are cost-effective and efficient [5,6,7,8]. For Lamb wave-based SHM algorithms, the key is to correctly tease out the signal features from the acquired wave signals and then associate them with the characteristics of damage (e.g., presence, location, and severity) [9]. However, this process faces many challenging problems because of the complex propagation characteristics of Lamb waves, including multimodal, dispersion, mode scattering, and conversion at irregularities. This drawback is particularly amplified and becomes a big challenge for damage identification in composite structures due to their material and geometric complexities.

For this reason, Lamb wave-based damage diagnosis is often achieved by measuring the differences in the wave responses between normal and faulty conditions [10,11,12]. Specifically, the initial waveforms taken in the undamaged condition are used as the baseline data, and any significant deviation in the new measurements beyond a threshold value indicates the presence of damage. However, it is susceptible to the operational and environmental conditions, especially the temperature variation [13,14,15]. To overcome this problem, the concept of time reversal process (TRP) has been explored in recent years. In the TRP, an input signal can be reconstructed at the source point if the signal received at another point is reversed in the time domain and emitted back to the source point [16]. When damage introduces nonlinearities, the time reversibility breaks down, and the discrepancies between the input signal and the TRP reconstructed signal indicate the presence of damage. Gangadharan et al. [17] investigated the time reversal behavior of Lamb wave modes and proposed a damage index (i.e., similarity coefficient) for damage detection. Through theoretical and experimental investigations, Wang et al. [18] found that it is impossible to reconstruct the waveform of the original excitation that consists of multiple frequency components because the time reversal operator is a function of frequency. Park et al. [19] developed a wavelet-based signal processing technique to extract Lamb wave response at the original input frequency value, and validated the feasibility of reconstructing the original input waveform by using a narrow-band excitation signal. To further improve its performance, Poddar et al. [20] investigated the influences of different parameters (e.g., frequency, bandwidth and transducer size) to the time reversal operator and the quality of the TRP reconstructed signal. Subsequently, Agrahari et al. [21] improved its sensitivity to damage by extending the signal length for computing the damage indices. Although the TRP has been extensively investigated for damage detection, there is still a lack of proper methods to compensate for the effects of time reversal operator.

In view of this issue, a modified time reversal method (MTRM) that could compensate the effects of time reversal operator and thus highlight the effects of damage is presented in this paper. First, the influences of time reversal operator to the final signal are theoretically analyzed. Subsequently, instead of applying an inverse filter to the final signal, the compensation of effects of time reversal operator is achieved by imposing a forward filter on the original input signal and measuring the discrepancies between the reconstructed signal and the modulated input signal. Once the effects of the time reversal operator are suppressed, the damages mainly determine the value of the modified damage index. Hence, the performance (i.e., reliability and applicability) of the time-reversed damage detection technique is improved. The rest of this paper is organized as follows. In Section 2, theories of the Lamb waves propagation in a composite plate are briefly reviewed. In Section 3, a modified time reversal method (MTRM) is proposed. Section 4 gives the experimental setups, and Section 5 illustrates the effectiveness of the MTRM for damage identifying by the experimental example. Finally, conclusions are drawn in Section 6.

## 2. Lamb Waves in a Composite Plate

Considering that a single-frequency Lamb wave is generated from a source at the coordinate origin in a composite plate, the wave arriving at the location **x** that is away from the source can be expressed as [22],

(1) $$u\left(t,x\right)=Ae^{j\left(\omega t-k\cdot x\right)}=Ae^{j\left[\omega t-k\left(\gamma \right)x\cos\beta \right]},$$

where *A* is the amplitude, *x* is the norm of **x**, *β* is the angle between the wave propagation, and wavenumber **k**, and *γ* is the angle of the wavenumber vector **k**.

Generally, the amplitude of a Lamb wave mode *A* is a function of frequency. In composite structures specifically, this frequency dependence is related to the excitability and detectability during the transmitting and receiving and the attenuation with the propagation of Lamb wave.

### 2.1. Excitability and Detectability

The excitability is defined as the ratio of displacement of a particular mode to force applied to the transmitter when both quantities are measured at the same location and direction in the cross section [23]. Generally, it is a function of frequency. For instance, if a piezoelectric ceramic (PZT) serves as a transmitter, the excitability function is related to the dimensions of the PZT and the fundamental properties of the plate [24].

The detectability would have the same value as the excitability when the receiving and transmitting transducers are exactly the same [23]. In this case, the amplitude *A*(*ω*) can be written as,

(2) A(ω)=T(ω)E(ω),

where *T*(*ω*) is the excitability and detectability of a Lamb wave mode, and *E*(*ω*) is the Fourier transform of the input signal.

### 2.2. Wave Attenuation

The definition of attenuation is related to the loss of wave amplitude as Lamb waves propagate through the structure. When the adjacent media is air, the attenuation due to dissipation into adjacent media is negligible. In this case, the wave attenuation mainly arises from the geometric spreading and material damping, and the amplitude *A*(*ω*) satisfies [25],

(3) $$A\left(\omega \right)=T\left(\omega \right)E\left(\omega \right)\frac{1}{\sqrt{x}}e^{-\eta \left(\theta ,\omega \right)x},$$

where, the term $1/\sqrt{x}$ is the attenuation due to the growing length of a wave front departing into all directions from a source (i.e., geometric spreading), while the term *e*^−*η*(*θ,ω*)*x*^ indicates the energy dissipation of a wave-packet due to non-perfect elastic material behavior (i.e., material damping). The attenuation coefficient *η* depends on the propagation direction *θ* and frequency *ω* [26]. It can be measured experimentally or predicted using different material models.

Hence, the system transfer function of a Lamb wave mode propagation in a composite plate can be expressed as,

(4) $$H\left(\omega ,x\right)=\frac{U\left(\omega ,x\right)}{E\left(\omega \right)}=T\left(\omega \right)\frac{1}{\sqrt{x}}e^{-\eta \left(\theta ,\omega \right)x}e^{-ik\left(\gamma ,\omega \right)x\cos\beta }.$$

## 3. Modified Time Reversal Method (MTRM)

The time reversibility (TR) of waves is fundamentally based on the linear reciprocity of the system [27,28]. If any source of nonlinearity exists along the wave path, the linear reciprocity and the TR break down. Thus, damages that introduce nonlinearities in the wave path could be detected by comparing the deviation between the reconstructed signal and the original input signal [19].

To implement the time reversal process for damage diagnosis, a transducer array is setup in a pitch-catch configuration. In each transmitter-receiver pair, to reverse the path direction, the transducer changes its role from transmitter to receiver and vice versa. The scheme of the time reversal method (TRM) is as follows [19],

(i) An input signal is emitted from transducer A, and the response is captured by transducer B;
(ii) The received signal is reversed in time domain;
(iii) The time reversed signal is reemitted from B, and the response (“reconstructed signal”) is recorded at A;
(iv) The reconstructed signal is time reversed and compared to the original input signal (both signals are normalized by their peak amplitudes).

For illustration, PZT A and PZT B are coupled to configure a transmitter-receiver pair. If an input signal *e*(*t*) is emitted from PZT A, the response recorded at PZT B could be viewed as a superposition of multiple Lamb wave modes. For a particular Lamb wave mode, its response can be represented by,

(5) U(ω,x)=H(ω,x)E(ω),

where *x* is the distance between PZT A and PZT B, *H*(*ω,x*) is the structural transfer function for the given signal path, and *E*(*ω*) is the Fourier transform of the input signal. In Fourier space, time reversal of the signal *U*(*ω,***x**) is defined as,

(6) $$U^{*}\left(\omega ,x\right)=H^{*}\left(\omega ,x\right)E^{*}\left(\omega \right),$$

where “*” denotes the complex conjugate of the function. If this time reversed signal is re-emitted by PZT B, the reconstructed signal captured at PZT A could be represented by,

(7) $$S\left(\omega ,x\right)=H\left(\omega ,x\right)U^{*}\left(\omega ,x\right)=H\left(\omega ,x\right)H^{*}\left(\omega ,x\right)E^{*}\left(\omega \right)=H_{TR}\left(\omega ,x\right)E^{*}\left(\omega \right),$$

Here, the time reversal operator *H*_TR_(*ω,x*)=*H*(*ω,x*)H^*^(*ω,x*)=|*H*(*ω,x*)|^2^.

Subsequently, the reconstructed signal is time reversed as,

(8) $$R\left(\omega ,x\right)=S^{*}\left(\omega ,x\right)=H_{TR}^{*}\left(\omega ,x\right)E\left(\omega \right),$$

Substituting Equation (4) into Equation (8), the final signal can be obtained as,

(9) $$\begin{array}{l} R\left(\omega ,x\right)={\left[T\left(\omega \right)\frac{1}{\sqrt{x}}e^{-\eta \left(\theta ,\omega \right)x}\right]}^{2}e^{-ik\left(\gamma ,\omega \right)x\cos\beta }e^{ik\left(\gamma ,\omega \right)x\cos\beta }E\left(\omega \right)\\ \;=\left[T^{2}\left(\omega \right)\frac{1}{x}e^{-2\eta \left(\theta ,\omega \right)x}\right]E\left(\omega \right) \end{array},$$

In time reversal method (TRM), the detection of damage is accomplished by measuring the discrepancy between this final signal and the original input signal. For instance, a damage index (*DI*) based on correlation coefficient is defined as [20],

(10) $$DI=1-\sqrt{{\left\{\int_{t_{0}}^{t_{1}}e\left(t\right)r\left(t,x\right)dt\right\}}^{2}/\left\{\int_{t_{0}}^{t_{1}}e{\left(t\right)}^{2}dt\int_{t_{0}}^{t_{1}}r{\left(t,x\right)}^{2}dt\right\}},$$

where *r*(*t*,*x*) is the inverse Fourier transform of *R*(*ω,x*), *t*_0_ and *t*_1_ define the time interval over which the signals are compared. Based on the definition, *DI* = 0 indicates the absence of any damage, while a non-zero value indicates the presence of damage.

As mentioned, in a composite plate, the excitability and detectability *T*(*ω*) and the material damping *e*^−*η*(*θ,ω*)*x*^ are generally dependent on the frequency. The frequency dependence of the time reversal operator *H*_TR_(*ω*,*x*) distorts the spectrum of the final signal and changes its waveform (Equations (8) and (9)). As a result, the main wave packet of the final signal cannot exactly match the original input waveform even in the undamaged condition. This deviation would also be captured by the *DI* (Equation (10)), arising a non-zero damage index and implying the existence of damage. Thus, the identification result worsens, and the risk of false alarm enlarges.

To overcome this problem and further improve the practical applicability of the time reversal concept, a compensation strategy is established as a key step of the modified time reversal method (MTRM). It is elaborated in the following steps.

Step 1: Estimation of amplitude of the transfer function (i.e., |Ĥ(ω,x)|)

In practical applications, Lamb wave propagation in a structure always consists of multiple modes. These modes are highly dispersive and quite possible to overlap with each other. To obtain accurate quantitative data on propagation characteristics, the Lamb wave mode of interest needs to be separated from the raw received Lamb wave signals.

Considerable research efforts have been directed toward the mode identification and separation. For instance, if a Lamb wave mode is separable in time domain, it could be extracted from the raw Lamb wave signal by a simple window function. Especially, for a dispersive Lamb wave mode, the dispersion compensation techniques could be applied to compress its time duration and enhance its separability [29].

If multiple Lamb modes overlap in time domain, time-frequency representation (TFR) may be an alternative as it provides a clear illustration for the temporal variation modal energy stream in the time-frequency domain [30]. Various improved TFR methods (e.g., matching pursuit [31], warped frequency transform [32], generalized warblet transform [33], adaptive Chirplet transform [34], and fractional Fourier transform [35]) have been developed for improving the time-frequency resolution and alleviating interaction of Lamb wave modes. On this basis, a Lamb wave mode may be extracted by time-varying filtering (e.g., Vold-Kalman filter) [34,36].

Without loss of generality, the circumstance where multiple Lamb wave modes interfere with each other in the time-frequency domain is also considered. In this case, if Lamb wave signals are recorded at a few discrete positions in the propagation direction, two-dimensional fast Fourier transform in time and space could map this two-dimensional recorded data set into the wavenumber-frequency domain [37]. In this new joint domain, the interfered Lamb wave modes may be separately identified, and mode extraction could be achieved by frequency-wavenumber filtering and multi-dimensional Fourier Transforms [38,39,40,41].

Once a Lamb wave mode *U*(*ω*,**x**) is separated, its transfer function could be calculated from Equation (4). Then, |*Ĥ*(*ω*,*x*)| could be obtained as the magnitude spectrum of the transfer function.

Step 2: Compensation of Frequency dependence

It can be observed from Equation (8) that the time reversal operator places a weighted factor at each frequency component of the final signal. Since it is a function of frequency, the wave components at different frequency values are non-uniformly scaled, and the shape of the final signal deviates from that of the input signal. Hence, a direct way to compensate its effects could be achieved by applying an inverse filter (i.e., *F_i_*(*ω,x*)=1/|*Ĥ*(*ω*,*x*)|^2^) to the final signal (i.e., Equation (8)),

(11) $$\tilde{r}\left(t,x\right)=\frac{1}{2\pi }\int_{-\infty }^{\infty }R\left(\omega ,x\right)F_{i}\left(\omega ,x\right)e^{j\omega t}d\omega =\frac{1}{2\pi }\int_{-\infty }^{\infty }\left[{\left|H\left(\omega ,x\right)\right|}^{2}/{\left|\hat{H}\left(\omega ,x\right)\right|}^{2}\right]E\left(\omega \right)e^{j\omega t}d\omega ,$$

However, this inverse filter is unstable because the estimated amplitude of the transfer function |*Ĥ*(*ω,x*)| is in its denominator. As a result, the compensated result would be quite sensitive to the noises in the final signal and the inaccuracies in |*Ĥ*(*ω,x*)|, which significantly restricts its practical applications.

According to Equation (10), the damage is detected by evaluating the discrepancies between the final signal and the original input signal. Rather than applying an inverse filter to the final signal, imposing a forward filter (i.e., *F_f_*(*ω,x*)=|*Ĥ*(*ω,x*)|^2^) on the original input signal may be a more robust alternative. In this way, the modulated input signal *ẽ*(*t*) could be obtained as,

(12) $$\tilde{e}\left(t\right)=\frac{1}{2\pi }\int_{-\infty }^{\infty }E\left(\omega \right)F_{f}\left(\omega ,x\right)e^{j\omega t}d\omega =\frac{1}{2\pi }\int_{-\infty }^{\infty }E\left(\omega \right){\left|\hat{H}\left(\omega ,x\right)\right|}^{2}e^{j\omega t}d\omega .$$

Subsequently, the damage index in Equation (10) is modified so as to evaluate the deviation between the final signal and the modulated input signal,

(13) $$MDI=1-\sqrt{{\left\{\int_{t_{0}}^{t_{1}}\tilde{e}\left(t\right)r\left(t,x\right)dt\right\}}^{2}/\left\{\int_{t_{0}}^{t_{1}}\tilde{e}{\left(t\right)}^{2}dt\int_{t_{0}}^{t_{1}}r{\left(t,x\right)}^{2}dt\right\}},$$

The amplitude of transfer function for a normal structure consists of two components, i.e., the excitability and detectability in the transmitting and receiving processes, and the attenuation with the propagation of Lamb wave mode (see Equation (4)). These factors are independent of damage. They influence the transfer function in both undamaged and damaged cases. In damaged cases, if they are compensated, the effects due to damage would be highlighted. In an ideal situation where the estimated amplitude of the transfer function exactly matches the actual one, the final signal in the undamaged case would exactly match the modulated input signal, and thus the smallest deviation caused by damage could be detected. It can be concluded that the effectiveness of compensation is determined by how accurately the amplitude of transfer function |*Ĥ*(*ω*,*x*)| can be estimated. On the other hand, if a defect is close to the plate edge, the sidebands related to the edge may more or less overlap the main wave packet (in the final signal). In this case, the accuracy of the damage index may also be determined by the time interval (*t*_0_, *t*_1_) over which the signals are compared.

In summary, the whole scheme of the MTRM is as follows,

(i) An input signal is emitted from PZT A, and an output signal is recorded at PZT B;
(ii) The output signal is reversed in time domain;
(iii) The time reversed signal is re-emitted from PZT B, and the reconstructed signal is recorded at PZT A;
(iv) The final signal is obtained as the time-reversed version of the reconstructed signal;
(v) The modulated input signal is calculated from Equation (12) by using the estimated amplitude of transfer function (in the undamaged case) as a prior knowledge;
(vi) Damage detection is accomplished by comparing the final signal to the modulated input signal (both signals are normalized by their peak amplitudes).

## 4. Experimental Setups

**Figure 1:**
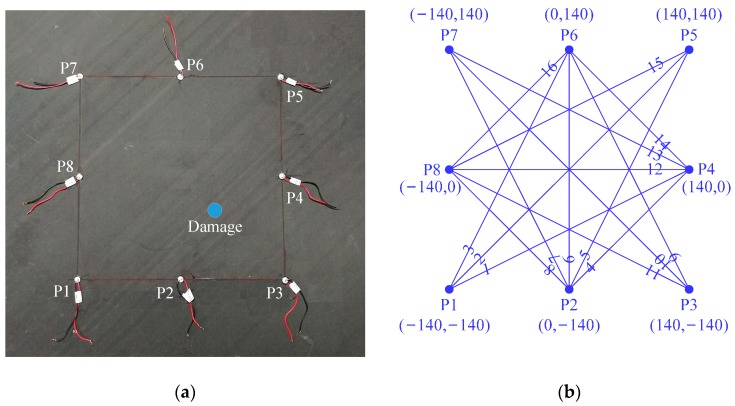
The CFRP laminate with (**a**) impact damage and (**b)** the coordinates and sensing paths of the sensing system.

The specimen under investigation is a T300/7901 16-ply carbon fiber-reinforced polymer (CFRP) laminate with the dimensions of 690 mm × 690 mm × 2 mm. The plate plies have the orientation [+45, −45, 0, 90, 90, 0, −45, +45]. The thickness of a single laminate is 0.125 mm, and the material properties are listed in Table 1. A series of spatially distributed PZTs (circular PZT, P51, 8 mm diameter and 0.5 mm thickness) are surface mounted on the specimen, as shown in Figure 1a. These PZTs are used as both transmitters and receivers to form an “active” local sensing system.

A coordinate system is then employed, where the center of the plate is set to be the coordinate origin and the monitoring area is spanned by the horizontal *x* and vertical *y* axes. Figure 1b gives the coordinates of the PZTs and the transmitter-receiver sensing paths of the sensing system. Referring to the literature [42], the quasi-static impact damage is imparted to the specimen using an indentation apparatus where a 20 mm diameter tup is mounted on a hydraulic jack equipped with a dynamometer. The damage is centered at (46, −46) mm and denoted as a blue-filled circle in Figure 1a.

The experimental setup consists of an Agilent 33120A waveform generator, an NF HSA4012 voltage amplifier, and an Agilent DSOX3014A oscilloscope. The waveform generator is used to generate a linear chirp signal where the frequency sweeps from 5 kHz to 1 MHz over a 1 ms rectangular window. The chirp signal is then amplified to 120 Vpp by the voltage amplifier and applied to the transmitter as the input signal. The oscilloscope is used for data acquisition of the response signals.

## 5. Results and Discussions

**Figure 2:**
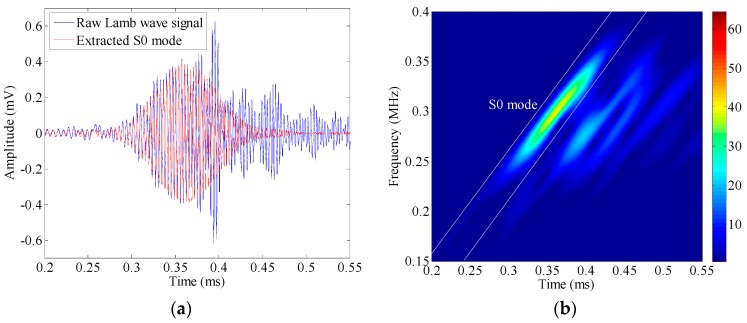
Comparison between the raw Lamb wave signal and the extracted S0 mode (**a**); and the short-time Fourier transform (STFT) spectrogram of the raw Lamb wave signal (**b**).

Sensing path PZT P1 to PZT P4 (i.e., Path 1 in Figure 1b) is discussed as an example. The Lamb wave signal captured before the introduction of the impact damage is shown in Figure 2a. With the application of short-time Fourier transform (STFT) where the window function takes a Gaussian function with the time duration of 56 μs, the time-frequency representation of the signal is calculated, and the associated spectrogram is depicted in Figure 2b. Since the excitation frequency is below the cut-off values of high-order Lamb modes, this signal only consists of the fundamental modes, i.e., S0 and A0. It is well acknowledged that in the low frequency range, S0 mode travels much faster than A0 mode. Hence, the first component in the spectrogram could be easily identified as the S0 mode transmitted from the source.

**Figure 3:**
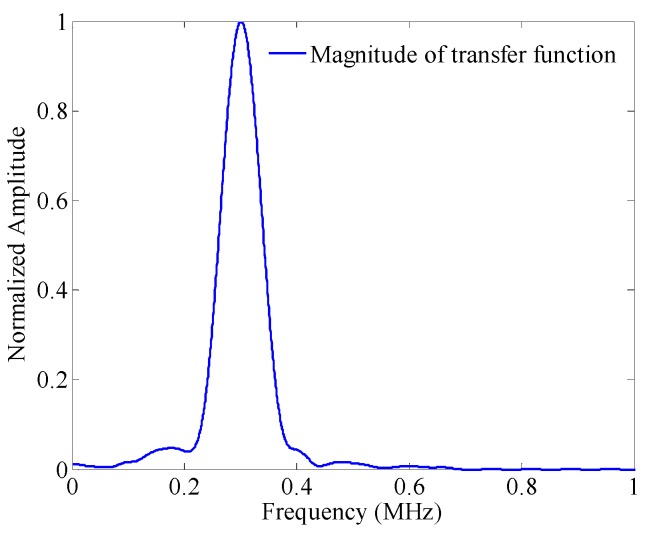
Normalized magnitude of transfer function of S0 mode.

As displayed in Figure 2b, the incident S0 mode is separable in the time-frequency domain. In this case, placing a tube into the spectrogram (the white lines in Figure 2b), setting all the points out of the tube to be zero, and then applying an inverse STFT, the S0 mode could be separated from the raw Lamb wave signal. Its waveform is displayed in Figure 2a as the red dashed line. The system transfer function associated with S0 mode is then estimated using the relation in Equation (4). Figure 3 shows the magnitude of the transfer function normalized by its peak value (at 301 kHz), as a function of frequency.

### 5.1. Identification Presence of Damage

**Figure 4:**
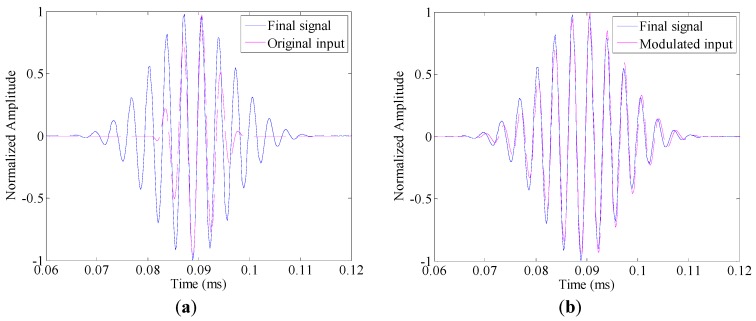
(**a**) Comparison between the final signal and the original input signal and (**b**) comparison between the final signal and the modulated input signal.

Five-cycle toneburst signals with their center frequency increases from 270 kHz to 330 kHz with a step of 20 kHz, are applied as the original input signals, respectively. The associated modulated input signals can be calculated from Equation (12) by applying the estimated amplitude curve of S0 mode |*Ĥ*(*ω*,*x*)| as a prior knowledge. For instance, when the center frequency takes 270 kHz, the original input signal and the modulated one are shown in Figure 4a,b, respectively. For comparison, the final signal obtained in the undamaged condition is also displayed in each figure. The modulated input signal matches the final signal well. In contrast, an obvious deviation between the original input signal and the final signal could be observed from Figure 4a.

**Figure 5:**
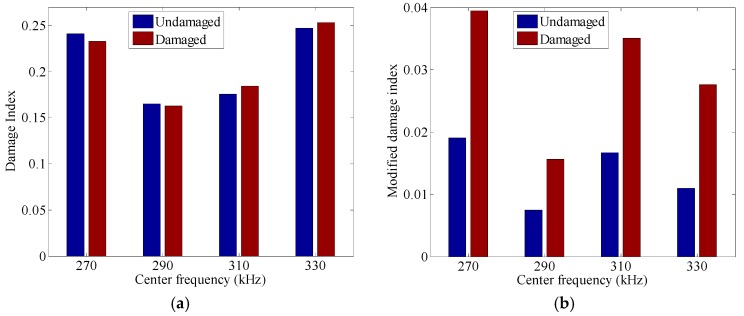
(**a**) The modified damage index in the modified time reversal method (MTRM) and (**b**) the damage index in the time reversal method (TRM) in both undamaged and damaged cases, where the original input signals are 5-cycle tonebursts with different center frequencies.

Subsequently, the TRM and the MTRM are employed in both undamaged and damaged cases. Figure 5 gives the damage index in the TRM and the modified damage index in the MTRM in these two different cases. In the TRM, the damage index evaluates the discrepancy between the final signal and the original input signal. After the damage is introduced, the nonlinearity along the wave path caused by the damage breaks down the time reversibility. Thus, wave distortion arises at the final signal. In this case, the deviation between the final signal and the original input signal may be attributed to two main factors, i.e., the time reversal operator and damage. However, even though both factors influence the waveform of the final signal and further the damage index, the interaction of them may not always leads to larger *DI* values. In practical applications, the damage index is always non-zero in the undamaged cases because of the measurement noises. Thus, in the time-reversal based damage detection technique, a threshold is needed to be pre-specified to distinguish the damaged cases from the undamaged ones [19]. Especially, if the DI value exceeds the pre-specified threshold value, the corresponding signal is defined as damaged. In this experiment, as shown in Figure 5a, when the center frequency of the toneburst signal takes 270 kHz and 290 kHz, the *DI* value in the damaged case is smaller than that in the undamaged case. It is impossible to set a threshold that is smaller than the *DI* value in the damaged case but larger than the one in the undamaged case. As a result, the TRM based damage detection technique cannot identify the presence of damage at these excitations.

In the MTRM, the effects of frequency dependence in the time reversal operator are compensated in the modulated input signal by Equation (12), and the modified damage index measures the discrepancy between the final signal and the modulated input signal. As shown in Figure 5b, in the undamaged case, the MDI in the MTRM reduces significantly when compared to the *DI* in the TRM, which ensures a much lower threshold for the undamaged case. In the damaged case, the MDI value at each excitation is much larger than that in the undamaged case. Hence, the MTRM could be employed at all excitations for damage detection.

Increasing the cycle number of a toneburst signal would narrow its frequency bandwidth and alleviate the effects of the time reversal operator [19]. In this experiment, toneburst signals centered at 270 kHz with the cycle number increases from 3 to 13 with a step of 2 are applied as the original input signals, for analyzing the influences of frequency bandwidth to the performance of time reversal methods. Table 2 lists the damage indices in the TRM and MTRM in both undamaged and damaged cases. The increment is obtained by subtracting the damage indices in the undamaged case from those in the damaged case. It measures the increase of damage indices with the presence of damage.

**Figure 6:**
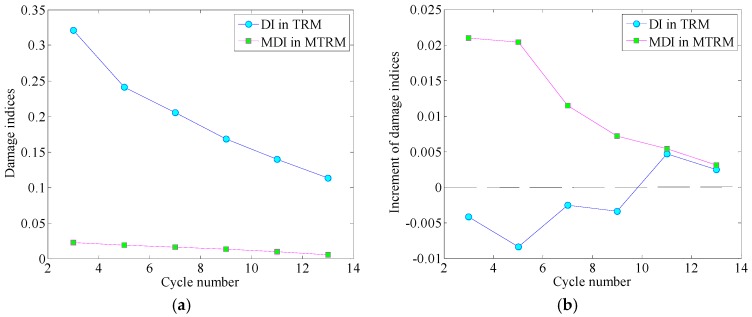
In undamaged case, (**a**) damage index in TRM and modified damage index in the MTRM decrease with the increase of cycle number, and (**b**) the evolution of sensitivities (to damage) with the increase of cycle number.

Figure 6a gives the evolution of damage indices (in the undamaged case) with the increase of cycle number. The damage index in the TRM decreases with the narrowing of the excitation frequency bandwidth. Similar phenomena could also be observed from the modified damage index in the MTRM. The reason could be explained as follows: the estimated magnitude of transfer function may be more or less different from the actual characteristic of the Lamb wave mode, and the effects of time reversal operator cannot be fully compensated in the MTRM. The residual effects, which are related to the frequency bandwidth, give rise to a non-zero modified damage index in the undamaged case. As the cycle number increases, the residual effects also weaken, thus the modified damage index decreases.

The sensitivities associated with the TRM and the MTRM under different excitations (toneburst signals with different cycle numbers) are demonstrated in Figure 6b. In the MTRM, the effects of the time reversal operator are greatly compensated. Thus, the damage dominates the discrepancies between the final signal and the modulated input signal and the increment of the modified damage index. Hence, the increment of the modified damage index remains above zero at each excitation. This increment decreases monotonously with the increase of cycle number, indicating that the sensitivity to the damage may weaken as the frequency bandwidth narrows. In comparison, the increment of damage index in the TRM is below zero when the cycle number is smaller than 11, indicating that the *DI* value in the damaged case is even smaller than that in the undamaged case. If the cycle number takes 11 and 13, the frequency dependence of time reversal operator may be weak enough that the effects of damage dominate the deviation of the final signal. As a result, the increment of the damage index goes beyond zero, and the TRM could identify the presence of damage under these excitations. However, as the cycle number further increases from 11 to 13, the increment of the damage index starts to decrease, which is similar to the phenomenon in the MTRM. Hence, it can be concluded that narrowing the excitation frequency bandwidth alleviates the frequency dependence of time reversal operator at the cost of weakening the sensitivity to the damage.

### 5.2. Damage Identification

To visually pinpoint structural damage, a probabilistic diagnostic algorithm is introduced, which estimates the probability of defect occurrence at any position (*x*,*y*) within the monitoring region as [10,11],

(14) $$P\left(x,y\right)=\sum_{k=1}^{N}P_{k}\left(x,y\right)=\sum_{k=1}^{N}I_{k}\left(\beta -R_{k}\left(x,y\right)\right)/\left(\beta -1\right),$$

where,

(15) $$R_{k}\left(x,y\right)=\frac{\sqrt{{\left(x_{k1}-x\right)}^{2}+{\left(y_{k1}-y\right)}^{2}}+\sqrt{{\left(x_{k2}-x\right)}^{2}+{\left(y_{k2}-y\right)}^{2}}}{\sqrt{{\left(x_{k1}-x_{k2}\right)}^{2}+{\left(y_{k1}-y_{k2}\right)}^{2}}},$$

*P_k_*(*x*,*y*) is the defect distribution probability estimated from the *k*th transmitter*-*receiver pair, *I_k_* is either the damage index or the modified damage index of this sensing path. (*x_k_*_1_, *y_k_*_1_) and (*x_k_*_2_, *y_k_*_2_) are the coordinates of the transmitter and the receiver of the *k*th path, respectively. *Β =* 1.05 is a scaling parameter. It is usually selected to be around 1.05 [10].

**Figure 7:**
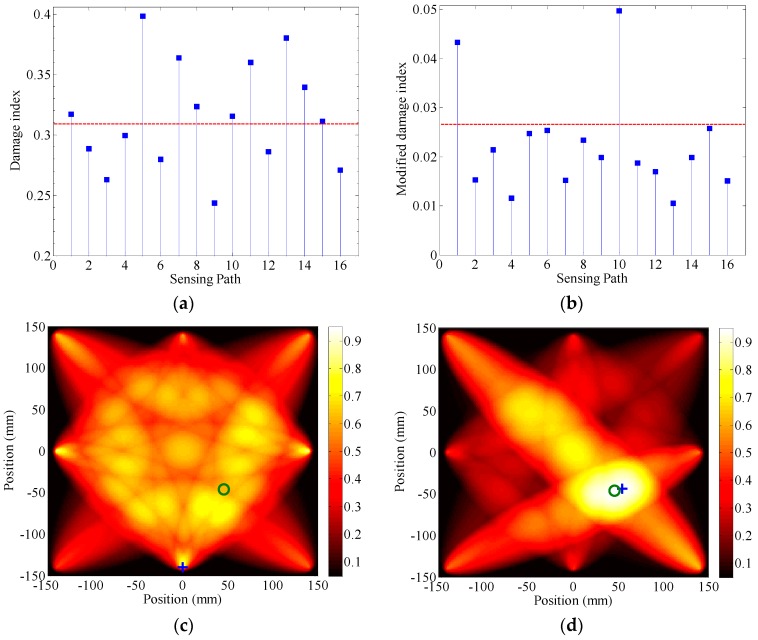
(**a**) Damage index of each transmitter-receiver pair in TRM, (**b**) modified damage index in the MTRM, (**c**) reconstructed images result from TRM, and (**d**) MTRM under a 3-cycle 270 kHz toneburst excitation.

In this section, a 3-cycle toneburst signal with a center frequency of 270 kHz is first applied as the original input signal. The values of damage index (in the TRM) of all possible sensing paths in the sensor network are displayed in Figure 7a. The *DI* values of sensing path 1 (i.e., P1–P4) and sensing path 10 (i.e., P3–P7) where the damage locates are even smaller than those of other paths that are isolated from the damage, e.g., sensing path 7 (i.e., P2–P7) and sensing path 13 (i.e., P4–P7). Hence, the presence of damage cannot be correctly identified. In comparison, the MDI values of sensing path 1 and sensing path 10 (in the MTRM) are obviously larger than all other paths, Figure 7b. Therefore, the damage could be identified as sitting at sensing path 1 and sensing path 10.

Subsequently, the *DI* values of all sensing paths in the sensor network are applied to Equation (14), mapping the structural damage to the tomogram. Figure 7c shows the reconstructed image associated with the TRM. The image is normalized by its maximum value. The actual location of the damage is marked by “o”. The grid with the highest probability value for the presence of damage, indicated by the small cross “+”, is considered as the center of identified damage. The tomogram is chaotic, thus the damage cannot be correctly identified. For comparison, the normalized reconstructed image associated with the MTRM is shown in Figure 7d. In this image, the grid with the highest probability value, (54, −44) mm, sits at the close neighbor of the damage location, (46, −46) mm, indicating that both the presence and location of the damage are accurately identified.

**Figure 8:**
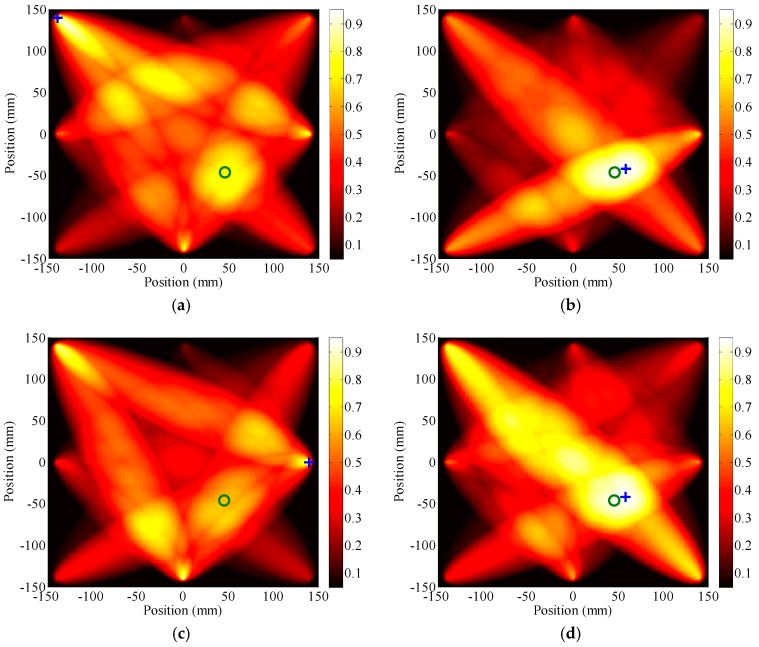
Reconstructed images result from TRM (the left hand side) and RTRM (the right hand side) corresponding to toneburst excitations with different cycle numbers: (**a**) and (**b**) 7 cycle; (**c**) and (**d**) 11 cycle.

For further illustration, toneburst signals with a center frequency of 270 kHz and the cycle number takes 7 and 11 are applied as the original input signal, respectively. Figure 8a,c shows the reconstructed images calculated from the TRM. In each image, the result is still fuzzy. It is nearly impossible to identify the numbers and locations of damages. The reason behind that is given as follows. When the cycle number becomes 7, the increment of damage index at the sensing path which across the damage area (e.g., sensing path P1–P4) is still below zero (see Table 2). Hence, the damage cannot be detected. As the cycle number increases to 11, the increment of damage index at sensing path P1–P4 goes beyond zero (Table 2). However, due to the differences in PZTs, sensing directions and path lengths, the frequency dependence of the time reversal operator would be distinct at different sensing paths. It is possible that the *DI* value of a sensing path isolated from the damage is larger than that of a sensing path crossing the damage area. In this case, the damage still cannot be correctly identified.

In comparison, Figure 8b,d shows the reconstructed images obtained from the sensor network using the MDI values calculated from RTRM. In both images, the damage is correctly identified. Besides, the identified damage locates at (58, −42) mm, which is close to its actual location. Hence, these illustrate a methodology with enhanced applicability and reliability for damage detection.

## 6. Conclusions

In this paper, a modified time reversal method (MTRM) is proposed for diagnostics of structural damages in a composite plate. The following conclusions can be drawn from the work:

(1) The frequency dependence of the time reversal operator is associated with the excitability and detectability, and the attenuation with propagation. It is independent of the damage, and could be compensated to improve the performance of the time-reversed damage detection;
(2) In the TRM, the deviation between the final signal and the original input signal may be attributed to both the frequency dependence of time reversal operator and the damage. In this case, the presence of damage may not always lead to an increase of damage index. Therefore, it may not be correctly identified;
(3) Narrowing the excitation frequency bandwidth alleviates the frequency dependence of the time reversal operator at the cost of reducing the sensitivity to damage;
(4) In the MTRM, the effects of frequency dependence of the time reversal operator are greatly compensated. A good waveform reconstruction can be achieved in the undamaged case, and the nonlinearity caused by the damage can be highlighted. Hence, it improves the reliability and applicability of the time-reversal based diagnostics;
(5) While attractive results are obtained, the effectiveness of the MTRM under the varying environmental and operational conditions deserves further investigation in the future work. As in references [43,44,45,46], conducting researches on the nonlinear behavior of the damage and capturing more information for a damage indicator is an important and interesting aspect for achieving a more robust damage detection.

## Figures and Tables

**Table 1:** Material properties of the carbon fiber-reinforced polymer (CFRP) plate.

Material Properties	*ρ* (kg/m^3^)	*E*_1_ (GPa)	*E*_2_ (GPa)	*G*_12_ (GPa)	*v*_12_	*v*_23_
Value	1560	135	10.9	4.7	0.285	0.4

**Table 2:** Damage index in TRM and modified damage index in MTRM in both undamaged case and damage case, as the toneburst signals take different cycle numbers.

Cycle Number	Damage Index in TRM			Modified Damage Index in MTRM		
	Undamaged	Damaged	Increment	Undamaged	Damaged	Increment
3	0.3212	0.3170	−0.0042	0.0223	0.0433	0.0210
5	0.2412	0.2328	−0.0084	0.0191	0.0395	0.0204
7	0.2057	0.2032	−0.0025	0.0159	0.0274	0.0115
9	0.1684	0.1650	−0.0034	0.0131	0.0203	0.0072
11	0.1398	0.1445	0.0047	0.0097	0.0151	0.0054
13	0.1129	0.1154	0.0025	0.0057	0.0088	0.0031
